# Development and validation of a method to quantify methylprednisolone aceponate in cream by micellar liquid chromatography

**DOI:** 10.1007/s00216-026-06380-x

**Published:** 2026-02-14

**Authors:** Elena Romero-Salicio, Juan Peris-Vicente, Diego E. Kassuha, Abhilasha Durgbanshi, Devasish Bose, Samuel Carda-Broch

**Affiliations:** 1 Department of Analytical Chemistry, Faculty of Chemistry, Universitat de València, 46100 Burjassot, Spain; 2 Instituto de Investigaciones en Ciencias Químicas, Facultad de Ciencias Químicas y Tecnológicas, Universidad Católica de Cuyo, San Juan, Argentina; 3 Department of Chemistry, Doctor Harisingh Gour Vishwavidyalaya (A Central University), Sagar, Madhya Pradesh 470003 India; 4 Department of Criminology and Forensic Science, Doctor Harisingh Gour Vishwavidyalaya (A Central University), Sagar, Madhya Pradesh 470003 India; 5 Department of Physical and Analytical Chemistry, ESTCE, Universitat Jaume I, 12071 Castelló, Spain

**Keywords:** Absorbance spectroscopy, Adventan, Corticosteroid, Eco-friendly, Validation

## Abstract

Adventan cream is a widely prescribed topical medication used to treat various skin disorders. We have developed a method based on micellar liquid chromatography to quantify its active component, methylprednisolone aceponate, which is an essential step for effective quality control. Samples were mixed with methanol and mobile phase to achieve a drug concentration of approximately 1 mg/L, filtered, and directly injected. Chromatographic analysis was performed in under 10 min with adequate resolution, using a mobile phase composed of 0.10 M sodium dodecyl sulfate—4.0%, v/v, 1-butanol, buffered at pH 3 with a phosphate salt, running at 1 mL/min under isocratic mode through a C18 column. Absorbance wavelength detection was set to 245 nm. The method was validated according to International Council for Harmonisation guidelines to assess its analytical performance in terms of specificity, instrumental linearity (*r*^2^ > 0.9996), calibration range (0.25 to 2.5 mg/L), limit of detection (0.09 mg/L), limit of quantification (0.26 mg/L), and robustness, as well as method precision (RSD < 2.8%) and trueness (relative recovery, 98–102.5%). It was also successfully applied to commercial samples. Additionally, the retention mechanism was investigated by determining the constants of the partition equilibria occurring in the column and demonstrating that both surfactant and organic solvent contribute similarly to elution strength. The method was safe and ecofriendly, due to the low volume of toxic and volatile organic solvents employed, easy to handle, cost-effective, semi-automated, and with a high sample throughput, making it well suited for routine pharmaceutical quality control.

## Introduction

Adventan cream (LEO Pharma, Ballerup, Denmark) is a highly effective, commonly used medication of external cutaneous use for the treatment of various inflammatory skin disorders, including eczema, allergic reactions, sunburn, atopic dermatitis, edema, and psoriasis. It alleviates symptoms, such as coarseness, pain, rash, dryness, redness, swelling, itching, exudation, burning sensation, and irritation of the skin. Its active component is methylprednisolone aceponate at a concentration of 0.1% (1 mg/g). Some of its excipients are decyl oleate, glycerol monostearate (40–50%), cetostearyl alcohol, hard fat, caprylic-capric-myristic-stearic triglycerides, polyoxyl 40 stearate, glycerol, disodium edetate, benzyl alcohol, butylhydroxytoluene, and water [1–4]. Adventan cream was approved for use in Spain in 1994 [1].

Methylprednisolone aceponate (MPA) is a fourth-generation, non-halogenated corticosteroid ester and glucocorticoid with potent anti-inflammatory activity. It penetrates the skin rapidly and undergoes swift metabolization and conjugation, resulting in low concentrations in serum. The medication exhibits an optimized efficacy/safety profile with minimal local or systemic adverse effects. Therefore, it offers the opportunity for once-daily application, which enhances patient adherence to treatment [2, 5–7].

**Fig. 1:**
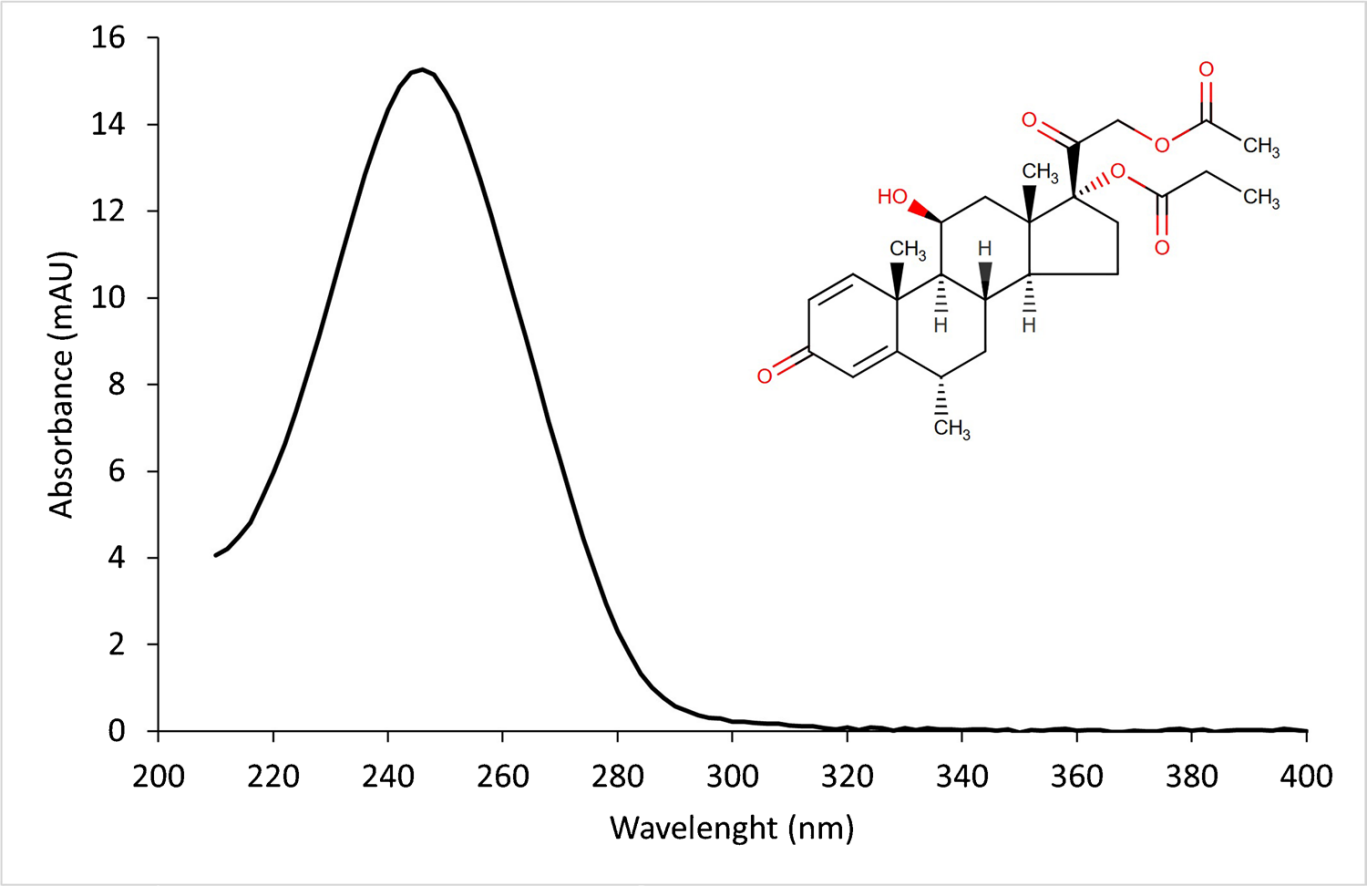
Structure of methylprednisolone aceponate and its absorption spectra taken at the retention time during elution using the selected conditions

Chemically, methylprednisolone aceponate, or [(6*S*,8*S*,9*S*,10*R*,11*S*,13*S*,14*S*,17*R*)−17-(2-acetyloxyacetyl)−11-hydroxy-6,10,13-trimethyl-3-oxo-7,8,9,11,12,14,15,16-octahydro-6H-cyclopenta[a]phenanthren-17-yl] propanoate, is a corticosteroid diester and a small molecule with a molecular mass of 472.6 g/mol. It is moderately hydrophobic with a log P_o/w_ of 2.9). It remains neutral in the pH interval 2 to 8, exhibiting no acidic alkaline activity [8, 9]. Its structure can be seen in Fig. 1 [9].

The determination of the active component in pharmaceutical dosage forms is a critical component of quality control during drug formulation and manufacturing. It also plays a key role in detecting counterfeit medications, ensuring therapeutic efficacy and safeguarding public health [10]. Currently, there is an urgent need for an analytical procedure to quantify MPA in cream formulations, particularly given the absence of an individual monograph for this drug in any official pharmacopoeia [11] and the limited number of methods reported in the scientific literature. MPA has been previously determined in creams using reversed-phase high-performance liquid chromatography coupled to ultraviolet absorbance detection (RP-HPLC-UV) [11], liquid chromatography coupled to mass spectrometry (LC-MS) [12], and liquid chromatography coupled to diode array detection and mass spectrometry (LC/DAD/MS) [13]. These methods typically employ a C18 column and mobile phases at an acidic pH, containing large volumes of organic solvents, like acetonitrile and/or methanol (often > 55%) to achieve proper resolution. Sample separation involves mixing the oily sample with large volumes of organic solvents, such as methanol/acetonitrile, methanol, or ethanol/acetone heating at 40ºC, followed by vigorous stirring and ultrasonication. The resulting supernatant is separated, filtered, and injected. However, the extensive use of toxic and volatile chemicals renders these procedures unsafe and pollutants. Besides, mass spectrometry is highly expensive and technically demanding, limiting its accessibility in many quality control laboratories. To address these limitations, we propose a greener and more cost-effective alternative based on hybrid micellar liquid chromatography-diode array detection (HMLC-DAD).

HMLC is a well-established branch of RP-HPLC, involving the use of C18 columns and surfactant solutions above the critical micelle concentration (CMC), thus assembled in micelles, and a low proportion of an organic solvent, as mobile phases and as solubilization or extractant solution during sample preparation [14]. The most commonly used surfactant is the anionic sodium dodecyl sulfate (SDS), which produces low-viscous solutions and is aerobically biodegradable and barely hazardous. As organic modifiers, 1-propanol, 1-butanol, and 1-pentanol, less hazardous than methanol or acetonitrile, are typically preferred [15]. Micellar solutions are effective at solubilizing oily matrices and small molecules with a wide range of polarity due to the interaction of hydrophobic groups with the micelle core. Consequently, a drug can be efficiently extracted from complex, high-lipid-content matrices such as creams and ointments through simple dispersion in micellar media followed by filtration [16]. The retention mechanism is also modified by the introduction of a secondary partition equilibrium for the solutes, between the bulk mobile phase and the micellar pseudophase. The elution strength of the mobile phase is then governed by both micelle concentration and the proportion of organic solvent, thus enhancing the versatility of the technique. The retention mechanism is highly stable and reproducible, and the retention factor can be predicted from the composition of the mobile phase using well-established chemometric models [17]. Compared to conventional hydroorganic methods, HMLC offers a greener and more easy-to-conduct alternative [18]. HMLC has been successfully used to determine corticosteroids in creams [19].

The goal of this study was the development of a green and easy-to-handle method to determine the corticosteroid methylprednisolone aceponate in a cream formulation by micellar liquid chromatography. The procedure was rigorously validated by the guidelines of the Validation of Analytical Procedures: Text and Methodology Q2(R1) from the International Council for Harmonisation to establish its analytical properties [20]. In addition, the retention mechanism was investigated by calculating the constants of the partition equilibria occurring in the column and assessing the influence of mobile phase composition on retention behavior. Finally, the method was applied to the analysis of commercial cream formulations.

## Experimental

### Standards and chemicals

Powdered solid standard of methylprednisolone aceponate (purity > 95.0%) was purchased from Merck KGaA (Darmstadt, Germany). Sodium dodecyl sulfate (> 99.0%) was bought from Tokyo Chemical Industry (Tokyo, Japan). Methanol (HPLC grade) and sodium dihydrogen phosphate (purity > 99%) were obtained from Sharlab (Barcelona, Spain), while 1-butanol was bought from Labbox (Barcelona, Spain). Hydrochloric acid (37%) was supplied by Panreac (Barcelona, Spain). Ultrapure water was in-lab prepared using an Adrona HPLC Connect purification system (Adrona, Riga, Latvia) which purified deionized water provided as tap water by the University. All aqueous solutions were prepared using this water.

### Preparation and solutions

To prepare the micellar solutions, appropriate masses of SDS and sodium dihydrogen phosphate were weighed and dissolved in water to obtain the desired concentrations (see sections "Chromatographic conditions" and "Optimization of the chromatographic conditions"). The pH was adjusted to 3 by adding drops of 1.0 M and 0.1 M HCl solutions. The solution was then transferred to a volumetric flask, and the required volume of 1-butanol was added to achieve the desired proportion. The mixture was diluted up to the mark with ultrapure water. Subsequently, the solution was ultrasonicated to ensure complete solubilization, filtered through a 0.45-µm Nylon membrane filter (Micron Separations) with the aid of a vacuum pump to remove particles, and poured into an amber bottle. The prepared micellar solutions were stored at room temperature in the dark.

A stock solution of methylprednisolone aceponate 100 mg/L was prepared by dissolving an accurately weighed amount of the standard in methanol. Working solutions were made by serial dilutions in mobile phase immediately prior to analysis. These solutions were not stored.

### Chromatographic conditions

The HPLC system consisted of an Agilent Technologies (Palo Alto, CA, USA) HP1100 equipped with a quaternary pump, a degasser, an autosampler, and a UV-Visible absorbance DAD. Agilent Chemstation software (Rev. B.04.03(16) 2010) was used to control the instrumentation, acquire and visualize chromatographic data, and calculate the basic chromatographic parameters, including dead time, retention time, peak area, and peak width. The tailing factor was calculated as per United States Pharmacopeia (USP) criterion [21].

Chromatographic separation was performed on a Kromasil C18 column without full end-capping (150 × 4.6 mm; 5 µm particle size; 10 nm pore size) purchased from AkzoNobel (Amsterdam, The Netherlands). The mobile phase consisted of a solution of 100 mmol/L sodium dodecyl sulfate-4% 1-butanol buffered at pH 3 with 0.01 M phosphate salt. The mobile phase was delivered isocratically at a flow rate of 1 mL/min. Injection volume and detection wavelength were 20 µL and 245 nm, respectively. Chromatographic instrumentation was operated according to maintenance protocols specific to micellar mobile phases [22]. All solutions to be injected were manually filtered through a 0.45-µm Nylon membrane filter (Micron Separations) using a 3-mL plastic syringe and introduced into the chromatographic vials, which were sealed and placed in the autosampler tray for analysis.

### Sample treatment

The study focused on the analysis of Adventan 1 mg/g cream, which was randomly bought from a local pharmacy and stored at +  4 °C in darkness.

Approximately 0.5 g of the cream and 10 mL of methanol were introduced in a beaker and hand-shaken to disperse the matrix. Subsequently, 30 mL of mobile phase was added, and the mixture was shaken by employing a magnetic stirrer for 15 min and ultrasonicated for 15 min, in order to enhance dispersion and solubilization of the matrix. The resulting solution was diluted up to 50 mL with mobile phase, yielding a methylprednisolone aceponate concentration of nearly 10 mg/L. This solution was further diluted 1/10 with mobile phase to obtain the target concentration of 1 mg/L, then filtered and directly injected. All sample solutions were discarded after analysis.

## Results and discussion

### Optimization of sample treatment

Cream is a semisolid viscous formulation composed of an emulsion of oily compounds and water, which is difficult to solve, even in micellar environments [3, 19]. Therefore, we aim to extract the active pharmaceutical ingredient (API) and achieve the maximum dispersion of the matrix.

A volume of 50 mL was used to disperse the samples, as it is the minimum volume that allows practical stirring with excessive solvent consumption. Methanol was chosen as the initial solvent to promote better dispersion. Sample masses of 0.5, 1, 2.5, and 5 g were tested. This last one produced excessive turbidity and was excluded. Among the others, the signal (normalized to sample mass) was similar for the 0.5 g and 1 g samples, but lower for the 2.5 g sample, indicating inefficient drug extraction at masses higher than 1.0 g. Ultimately, 0.5 g was selected due to its smaller turbidity, because there was not any sensitivity issue. Lower masses were not tested because uncertainty during sampling would be too high. The resulting suspension was filtered to prevent particle introduction in the column. A volume of 2 mL was easily obtained without clogging.

The sample treatment involves only an easy-to-handle solubilization step in a solution containing less than 25% of organic solvent, without any purification or extraction phase. Besides, the procedure can be semi-automated, and many samples can be simultaneously processed.

### Optimization of the chromatographic conditions

The general chromatographic conditions were selected from previous works on drug determinations in pharmaceutical formulations [23].

MPA does not exhibit acidic or basic properties and remains neutral within the operating pH range of the column (2.5–7.5). The pH was set to 3, to ensure that the free silanol groups on the stationary phase remain neutral, rather than anionic. This helps prevent charge-dipole and charge-induced dipole interactions between the deprotonated free silanols (pKa4.0 - 5.0) and the drug molecules [24]. MPA has moderate hydrophobicity (log P_o/w_ = 2.9) and contains some polars groups. As excessive retention was not expected, 1-pentanol was excluded because it would provide an excessive elution strength to the mobile phase. Instead, 1-butanol was selected as the organic solvent to enhance the elution strength of the mobile phase and improve peak shape. Optimization of SDS and 1-butanol concentrations was carried out using a sample processed to get an injected solution of 10 mg/L MPA, since possible interfering compounds have to be considered at this stage. This solution was analyzed using five different mobile phase composition, selected by a full factorial design plus the central value. The −1 and +1 levels corresponded to the minimal and maximal concentrations typically recommended for HMLC [25]. The assayed mobile phase compositions (expressed as SDS, mmol/L/1-butanol, %v/v) and the obtained retention times (min) for MPA were: 50/1, 20.00; 50/7, 10.11; 100/4, 6.24; 150/1, 10.00 and 150/7, 4.14. In all cases, a second high peak, eluting between the dead time and that of MPA was observed. However, it did not interfere with the analysis.

MPA displays a micelles-binding behavior, as evidenced by the decrease of retention time at higher concentrations of SDS. The operating mobile phase was selected from those previously tested. Mobile phases that resulted in retention times longer than 10 min were excluded. Conversely, the mobile phase with the highest elution power was also ruled out due to its high backpressure, elevated salt content (increasing the risk of columns clogging), and excessive organic solvent volume. Consequently, the selected mobile phase was an aqueous solution of 100 mM SDS, 4% 1-butanol, buffered at pH 3 with 0.01 M phosphate salt. Under these conditions, the analyte was eluted in a short time (less than 10 min), without coelution with the solvent front or with excipients eluting at higher retention times. This separation was achieved under isocratic conditions using a mobile phase with < 5% of a volatile and toxic organic solvent, significantly lower than the levels typically required in hydroorganic RP-HPLC. This represents a noteworthy accomplishment, considering the complexity and viscosity of the matrix, as well as the absence of any purification step.

To determine the optimal detection absorbance wavelength, a standard solution of MPA 10 mg/L was analyzed under the selected separation conditions, and the UV absorbance spectrum was recorded at the retention time (Fig. 1). A strong absorption band was observed between 210 and 290 nm, with a maximum at 246 nm. This wavelength was therefore chosen for the method. The obtained spectrum was consistent to that reported in [26], indicating that the micellar environment has a limited impact on absorbance spectroscopic properties of MPA. Additionally, the UV absorbance spectrum was also measured at 5%- and 50%-height front and tail of the chromatographic peak to support subsequent peak purity assessments.

System suitability testing was conducted by performing six replicate injections (*n* = 6) of the processed sample at 10 mg/L. The experimentally determined dead time was 0.9 min, taken as the first elevation of the baseline. The evaluated parameters and their corresponding acceptance criteria [21] were retention time, 6.24 ± 0.07 min (within acceptable range); relative standard deviation (RSD%) of retention time, 1.2, < 2.0; standard deviation of peak area, 0.7, < 2.0; retention factor, 5.9, > 2.0; column efficiency as number of theoretical plates, 2351, > 2000, and tailing factor, 1.2 (0.8–1.6). These results demonstrated that the chromatographic system, under the selected conditions, provides reliable and consistent performance suitable for the analysis of MPA in cream samples.

### Theoretical study on retention

In HMLC using C18/SDS, the retention mechanism is governed by the three-phase theory. Retention depends on the partition equilibria of the solute between the bulk mobile phase and the SDS-modified stationary phase (similar to RP-HPLC) and between the bulk mobile phase and the micellar pseudophase (secondary interaction) [27]. These interactions are influenced by the physicochemical properties of the solute and the composition of the mobile phase. In this study, we focus on the effects of the surfactant and organic modifier at a fixed pH of 3.

The quantitative influence of SDS and 1-butanol on the retention factor was determined using an empirical model (Eq. 1), which provides accurate results for moderately hydrophobic solutes [28]:

1 $$\frac1k=c_0+c_1\left[\mathrm{SDS}\right]+c_2\left[1-\mathrm{butanol}\right]+c_{12}\left[\mathrm{SDS}\right]\;\left[1-\mathrm{butanol}\right]$$

The equation was fitted with the experimental data obtained by applying the experimental design described in section “Optimization of the chromatographic conditions,” with the concentrations normalized within the range −1 and +1. In this context, *c*_0_ represents the elution strength of the mobile phase under intermediate conditions, while *c*_1_, *c*_2_, and *c*_12_ correspond to the effect of SDS concentration, organic modifier proportion, and their interaction, respectively. A reasonable goodness-of-fit was achieved, with a multiple coefficient of determination (*R*^2^) of 0.96 and a maximum error of prediction of 22% (1 degree of freedom). The model parameters were as follows: *c*_0_ = 0.138 ± 0.015; *c*_1_ = 0.058 ± 0.017; *c*_2_ = 0.058 ± 0.017; and *c*_12_ = 0.032 ± 0.017. Both compounds have a moderate and comparable influence on the elution strength, while their interaction was slightly significant.

The same model can be applied to calculate the partition constants associated with the various equilibria involved in the retention process. Equation (1) can thus be reformulated as the mechanistic Eq. (2), where the constants have a defined physicochemical interpretation [28]: 

2 $$k=\frac{{FK}_{AS}{\displaystyle\frac1{1+K_{AD}\left[1-butanol\right]}}}{1+K_{AM}\frac{1+K_{MD}\left[1-butanol\right]}{1+K_{AD}\left[1-butanol\right]}\left[SDS\right]}$$

This equation was fitted with the experimental data obtained in the assay described in “ Optimization of the chromatographic conditions,” using the original unities. In this context, *F* represents the phase ratio and *K*_AS_ and *K*_AM_ denote the constants for partition equilibria of the solute between the bulk mobile phase, and the stationary phase and between the micelle, respectively, in the absence of organic solvent. *K*_AD_ and *K*_MD_ quantify the increase in solubility, and thus the displacement of the equilibria towards the mobile phase and the micelle, respectively, upon addition of the organic modifier. Accordingly, retention increases at higher values of *K*_AS_ and decreasing values of *K*_AM_, *K*_AD_, and K_MD_. The model achieved the same goodness-of-fit as Eq. (1), with the following results: *FK*_AS_ = 32.14; *K*_AM_ = 9.77; *K*_AD_ = −0.073; and *K*_MD_ = 0.70.

According to these results, MPA displays moderate-to-strong union to the SDS-modified stationary phase and moderate-to-weak to the micellar pseudophase, primarily through hydrophobic interactions (the analyte is hydrophobic and neutral), and the hydrophobicity of the stationary phase is far higher than that of the micellar pseudophase. Interestingly, 1-butanol slightly diminishes the solubility of MPA in the bulk mobile phase, an unexpected observation. Conversely, 1-butanol seems to enhance the interaction of the solute with the micelles. This effect is likely due to a reduction in the CMC and a subsequent increase in the number of micelles, as alcohol monomers incorporate into the micelle structure. Overall, these findings suggest that the solubility of MPA in the micellar solution, and consequently in the elution strength, is essentially governed by the interaction with the micelles, rather than with the organic solvent.

### Method validation

This procedure was validated in accordance with the guideline “Validation of Analytical Procedures: Text and Methodology Q2(R1)”, published by the International Council for Harmonisation [29] as well as the “FDA-ORA Laboratory Manual, Volume II – Methods, Method Verification, and Validation”, from the US Food and Drug Administration—Office of Regulatory Affairs [30].

#### Calibration range and linearity

Five standard solutions of MPA, ranging from 0.25 to 2.5 mg/L, were prepared and analyzed (*n* = 6). The homoscedasticity of the model was assessed using a two-tailed *F*-test (confidence level of 0.05) comparing the highest and lowest variances. As homoscedasticity was confirmed, least-squares linear regression was applied to establish a mathematical relationship between the concentration (independent variable) and the peak area (dependent variable). The limit of detection (LOD) and the limit of quantification (LOQ) were calculated as 3.3 and 10 times the standard deviation of the y-intercept (taken as the blank) divided by the slope (sensitivity).

The results for instrumental slope, *y*-intercept, determination coefficient, relative residual standard deviation, LOD, and LOQ were 33.0 ± 0.8, −2.7 ± 0.9, 0.9996, 2.0%, 0.09 mg/L, and 0.26 mg/L. The model showed excellent goodness-of-fit and linearity, with *r*^2^ and relative residual standard deviation > 0.995 and < 3.0%, respectively. Zero was included in the confidence interval of the y-intercept (confidence level of 5%), indicating the systematic error was negligible. The validated linear range (0.25–2.5 mg/L) covers 80–120% of the target concentration.

For the method, the linear range was 0.25–2.5 mg/g, with an LOD of 0.09 mg/g and a LOQ of 0.26 mg/g.

#### Specificity

**Fig. 2:**
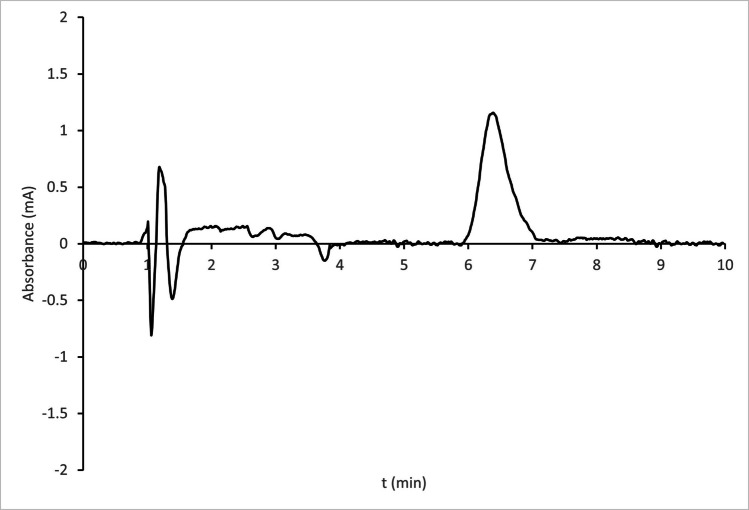
Chromatogram obtained by the analysis of a standard solution of MPA 1 mg/L

**Fig. 3:**
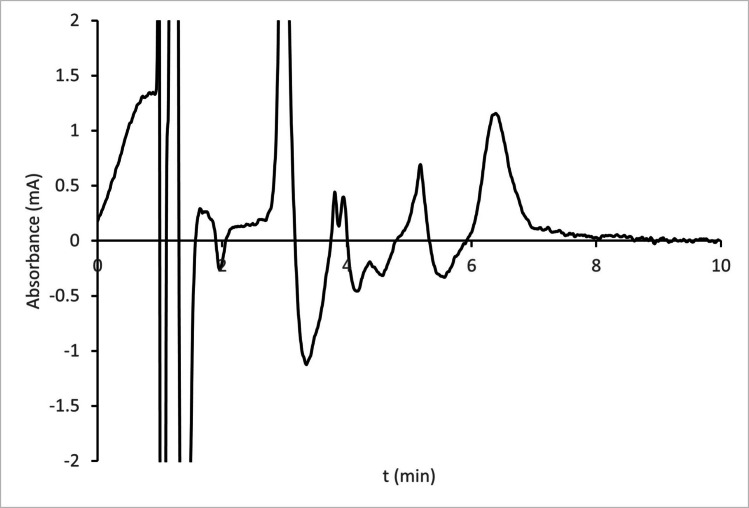
Chromatogram obtained by the analysis of an Adventan sample processed to get an injected solution of 1 mg/L

The ability of the procedure to distinguish the drug from excipients, impurities, and degradation products was evaluated by comparing chromatograms obtained from a standard solution of MPA 1 mg/L (Fig. 2) and a sample processed and diluted as described in section “Sample treatment” to get an injected solution of MPA 1 mg/L (Fig. 3).

The sample chromatogram displays a front from the dead time to 2 min, followed by a sharp and intense peak at approximately 4 min, along with several small peaks. None of them overlapped with the analyte. When both chromatograms were overlaid, the analyte peaks were found to closely match in terms of shape, area, and retention time.

To assess peak purity, UV absorption spectra were collected at the analyte’s retention time, as well as at 50% and 5% height on the front and tail of the peak in both chromatograms. First, the five spectra obtained from the processed sample were overlaid and found to be highly similar, with only the 5% spectra exhibiting slightly increased noise. Next, each spectrum from the sample was compared to its corresponding spectrum from the standard solution; all were consistent.

These results confirm the absence of co-eluting compounds in the matrix and demonstrate that the method is specific to MPA.

#### Trueness and precision

These parameters were determined at 80%, 100%, and 120% of the target concentration using the standard addition method.

At each level, six aliquots of a cream sample were processed to obtain an injected solution containing 0.5 mg/L and spiked with the studied level. Each fortified sample was analyzed before and after fortification, and the found concentration was calculated by difference. Trueness was expressed as the ratio of the found concentration to the true value (relative recovery), while repeatability was the relative standard deviation (RSD) of the results. This procedure was repeated over five different days during a 3-month period, using freshly prepared solutions. Intermediate precision was assessed as the RSD of the 30 found concentrations. The results are summarized in Table 1.

The method provides reliable and consistent quantitative results, all within the acceptance criteria, due to the solubilization capacity of micellar solutions and the absence of an extraction or purification step. Furthermore, systematic replicate injections for all samples are not required, resulting in significant savings of both effort and resources. However, replicate injections can be introduced for some of the samples to ensure consistency of the results during the sequence run.

**Table 1:** Results obtained for trueness and precision

Concentration (mg/L)	Trueness (relative recovery, %)	Repeatability (RSD, %)	Intermediate precision (RSD, %)
0.8	102.4	2.0	2.8
1.0	98.1	1.8	2.9
1.2	100.6	1.9	2.6
Acceptance criteria	97–103%	< 2.0	< 3.0

#### Cross-contamination

A sample was processed to obtain an injected solution of 5 mg/L, and a blank (pure mobile phase) was successively analyzed. The chromatogram of the blank showed no peaks or baseline disturbances within the analyte’s window time, indicating that cross-contamination was insignificant at the working concentration levels. Nevertheless, it was decided to include a blank injection before each sample injection to enhance the robustness of the method.

#### Robustness

Small variations in the main instrumental conditions that can commonly occur in the usual work at the laboratory were deliberately introduced to evaluate their effect on the instrumental responses (retention time and peak area), following a one-factor-at-a-time approach. For each parameter, a standard solution of 1 mg/L MPA was analyzed at the optimal value and at the minimum and maximum values within a predefined oscillation range, while the other parameters were held constant at their optimal value. Robustness was assessed based on the RSD of these three measurements. The experimental parameters and their respective variation ranges were SDS concentration (± 0.01 M), proportion of organic solvent (± 0.2), pH (± 0.2), detection wavelength (± 5 nm), and flow rate (± 0.05 mL/min).

The method was considered sufficiently robust, as all the RSD values were < 5.0%, indicating minimal impact from these minor fluctuations. Nonetheless, careful control of them is recommended to ensure optimal response stability.

### Greenness evaluation

The greenness of the method was evaluated using the MoGAPI assessment tool [31]. This tool offers a holistic, reliable, and robust assessment of method greenness by presenting 16 questions covering various aspects of the procedure (sample preparation, reagents and solvents, instrumentation, and quantification). Each question has multiple predefined responses, with assigned credit values; the greener the response, the higher the credit. The total score is then calculated as a percentage of the maximum possible credits. Additionally, the tool provides visual feedback through red/green/yellow pictograms for each question and assigns an overall color rating to help identify both strengths and weaknesses of the method at a glance.

**Fig. 4:**
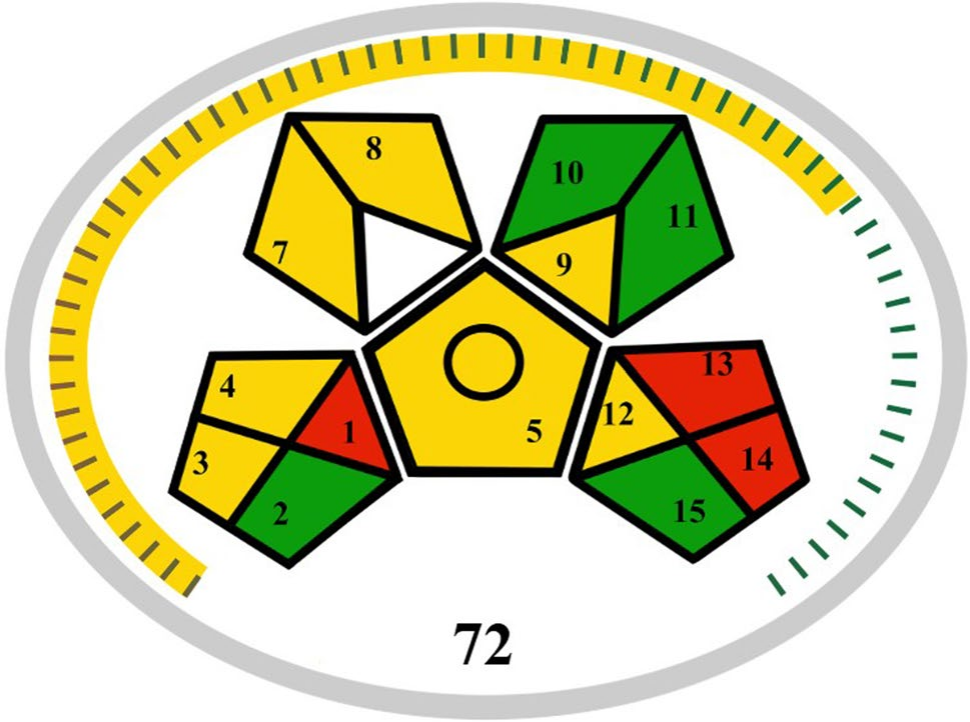
Pictogram obtained by greenness assessment using the MoGAPI tool

The evaluated method achieved a score of 72/100 (yellow), indicating a fair and acceptable level of greenness. The pictogram can be seen in Fig. 4. The main limitations identified were the separation of the sampling, treatment, and signal measurement steps, as well as the absence of miniaturization.

### Analysis of commercial samples

The suitability of the procedure for routine analysis was assessed using a cream formulation purchased from a local pharmacy. The sample was analyzed in duplicate, and quality control (QC) standards as well as blanks were included in the same run to ensure data consistency and to confirm the absence of cross-contamination during the analysis.

The following solutions were prepared and successively injected in the same sequence run (result): QC 1 mg/L (0.96 mg/L), blank (not detected), Adventan 1 mg/g (label claim, 97.8%), QC 2 mg/L (1.97 mg/L), blank (not detected), Adventan 1 mg/g (label claim, 99.5%), blank (not detected), QC 0.5 mg/L (0.52 mg/L). Only one solution was prepared, as the mobile phase was used both to prepare standard solutions and achieve the solubilization of the samples. The total analytical run time for each sample was nearly 40 min, but several samples could be simultaneously processed.

The MPA peak was clearly identified without any interference. Found concentration for QCs was around the nominal ones, and MPA was not detected in any of the blank injections, confirming the consistency and reliability of the responses provided by the instrumentation. Furthermore, replicate analyses of the sample yielded similar results. Finally, the pharmaceutical preparation was found to be compliant with regulatory requirements, as the label claim was 95–105%. 

The procedure was user-friendly, semi-automated, and allowed for the analysis of multiple samples within a single day. Mainly harmless and biodegradable reagents were employed, with the exception of the solvents methanol and 1-butanol. However, these solvents were used in significantly lower quantities compared to typical hydroorganic HPLC methods. Moreover, only globally available generic chemicals, instrumentation, and laboratory resources were required.

## Conclusions

Hybrid micellar liquid chromatography has proven to be a valuable technique for the determination of methylprednisolone aceponate in cream formulations. Its main advantages include the simplicity of the sample treatment and the use of solutions containing minimal volumes of hazardous organic solvents, owing to the solubilization power of micellar systems. The method was successfully validated following the guidelines of the ICH, thereby proving the accuracy and consistency of the results, and was effectively applied to the analysis of commercial samples. What’s more, the procedure was easy to conduct, safe, ecofriendly, cost-effective, and capable of a high sample throughput, making it well suited for routine analysis in the pharmaceutical industry.

An interpretative approach based on chemometrics was conducted for a theoretical investigation of the retention mechanism, using a limited number of experimental assays, to explore the behavior of the solute within the chromatographic column. The study revealed the influence of surfactant and 1-butanol on the drug’s retention factor, as well as the value of the partition constants associated with the equilibria governing the retention process.

## Data Availability

Data will be made available on request.
